# Exploring the Challenges of Lipid Nanoparticle Development: The In Vitro–In Vivo Correlation Gap

**DOI:** 10.3390/vaccines13040339

**Published:** 2025-03-21

**Authors:** Sarah Lindsay, Muattaz Hussain, Burcu Binici, Yvonne Perrie

**Affiliations:** Strathclyde Institute of Pharmacy and Biomedical Sciences, University of Strathclyde, 161 Cathedral Street, Glasgow G4 0RE, UK; sarah.lindsay@strath.ac.uk (S.L.); muattaz.y.hussain@strath.ac.uk (M.H.); burcu.eryilmaz@strath.ac.uk (B.B.)

**Keywords:** lipid nanoparticles, LNPs, mRNA, in vitro, in vivo, vaccines, expression

## Abstract

Background/Objectives: The development of lipid nanoparticles (LNPs) as delivery platforms for nucleic acids has revolutionised possibilities for both therapeutic and vaccine applications. However, emerging studies highlight challenges in achieving reliable in vitro–in vivo correlation (IVIVC), which delays the translation of experimental findings into clinical applications. This study investigates these potential discrepancies by evaluating the physicochemical properties, in vitro efficacy (across three commonly used cell lines), and in vivo performance (mRNA expression and vaccine efficacy) of four LNP formulations. Methods: LNPs composed of DSPC, cholesterol, a PEGylated lipid, and one of four ionizable lipids (SM-102, ALC-0315, MC3, or C12-200) were manufactured using microfluidics. Results: All formulations exhibited comparable physicochemical properties, as expected (size 70–100 nm, low PDI, near-neutral zeta potential, and high mRNA encapsulation). In vitro studies demonstrated variable LNP-mediated mRNA expression in both immortalised and immune cells, with SM-102 inducing significantly higher protein expression (*p* < 0.05) than the other formulations in immortalised and immune cells. However, in vivo results revealed that ALC-0315 and SM-102-based LNPs achieved significantly (*p* < 0.05) higher protein expression without a significant difference between them, while MC3- and C12-200-based LNPs exhibited lower expression levels. As vaccine formulations, all LNPs elicited strong immune responses with no significant differences among them. Conclusions: These findings highlight the complexities of correlating in vitro and in vivo outcomes in LNP development and demonstrate the importance of holistic evaluation strategies to optimise their clinical translation.

## 1. Introduction

Lipid nanoparticles (LNPs) play a key role in the delivery of mRNA vaccines. Their ability to encapsulate and protect fragile nucleic acid cargo, such as mRNA and siRNA, while facilitating cellular uptake and endosomal escape, has positioned LNPs as the leading delivery system for mRNA vaccines. This is evidenced by the rapid development and deployment of mRNA-based vaccines against SARS-CoV-2 (Comirnaty^®^ and Spikevax™ [1]) and respiratory syncytial virus (RSV) (mRESVIA^®^), which demonstrates the scalability, safety, and efficacy of LNP formulations in clinical settings.

Advancements in LNP design have focused on optimising their composition, which typically includes an ionizable lipid, a phospholipid, cholesterol, and a PEGylated lipid. These components synergistically enhance the stability, cellular delivery, and biocompatibility of LNPs. Additionally, innovations in LNP manufacturing technologies, such as microfluidic techniques, have enabled precise control over particle size and reproducibility, further enhancing the development and clinical application of LNPs.

The performance of LNPs for mRNA delivery is dependent on their potency, in vivo distribution and targeting, ability to induce an immune response, and inherent adjuvanticity [2]. Cellular uptake of these LNPs is a multi-step process mediated by the LNP’s size and the interaction between LNPs and biological fluids, specifically biomolecules such as immunoglobulins and lipoproteins [3]. LNPs can be internalised by several methods, including micropinocytosis and clathrin-mediated endocytosis, with the endocytic pathway dependent on the nanoparticle properties and the type of cells [4]. After entering the cells, the LNPs enter the endosome, where the ionisable lipid becomes protonated due to the lower, acidic pH in the endosomal compartment. This positive charge on the ionisable lipid then allows for interaction with the negatively charged phospholipids within the endosome, thereby facilitating endosomal escape and release of the mRNA payload into the cytosol, where it is translated [1].

Developing LNPs requires a series of stages, from formulation design to manufacturing optimisation and preclinical testing. Preclinical evaluation of LNPs typically begins with their physicochemical characterisation (including particle size, polydispersity index (PDI), zeta potential, and mRNA encapsulation. These characteristics are often defined as critical quality attributes (CQAs) for LNPs and can determine LNP quality, safety and efficacy. Following characterisation, in vitro studies evaluate LNP performance in controlled cellular environments. These studies focus on cellular uptake efficiency, transfection potential, and the ability of LNPs to escape endosomes and deliver their nucleic acid cargo to target sites. Immortalised and immune cell lines are commonly used to provide insights into the interactions between LNPs and different cell types. Transfection efficacy, often measured through protein expression assays, is a key metric for assessing in vitro performance. The next phase of development involves in vivo studies, which are critical for understanding LNP behaviour and performance in complex biological systems. These studies focus on biodistribution, protein expression levels, and vaccine efficacy, providing insights into how LNPs deliver mRNA to specific tissues and initiate an immune response.

Preclinical studies aim to establish a predictive framework that correlates in vitro results with in vivo outcomes, enabling researchers to forecast clinical performance accurately and efficiently. Establishing a reliable IVIVC for mRNA-LNP vaccines would significantly aid the formulation of LNPs across all stages of development. In the early stages, IVIVC can streamline formulation optimisation by predicting in vivo performance from in vitro data, reducing the reliance on extensive animal testing. In clinical development, IVIVC enables more effective translation of preclinical findings to human trials, reducing uncertainties in therapeutic outcomes. However, establishing IVIVC for LNPs is a critical yet complex challenge. For instance, Paunovska et al. used high-throughput DNA barcoding to compare the in vitro and in vivo delivery of hundreds of LNPs to endothelial cells and macrophages [5]. They found that in vitro delivery did not predict in vivo delivery. Similarly, Escalona-Ryao et al. showed divergent in vitro and in vivo results for mRNA-LNP formulations that used clinically approved ionizable lipids (Dlin-MC3-DMA, ALC-0315 and SM-102) [6]. In their studies, the authors looked at in vitro expression in dendritic cells, macrophages, and primary (BMDCs) immune cells. They then compared this to protein expression levels in zebrafish embryos and intracellular cytokine responses produced by antigen-specific T cells in mice after a single-dose subcutaneous administration of the mRNA-LNPs. The authors concluded that the ionizable lipid modulates the performance of mRNA-LNPs and that in vitro data does not adequately predict their behaviour in vivo.

**Figure 1:**
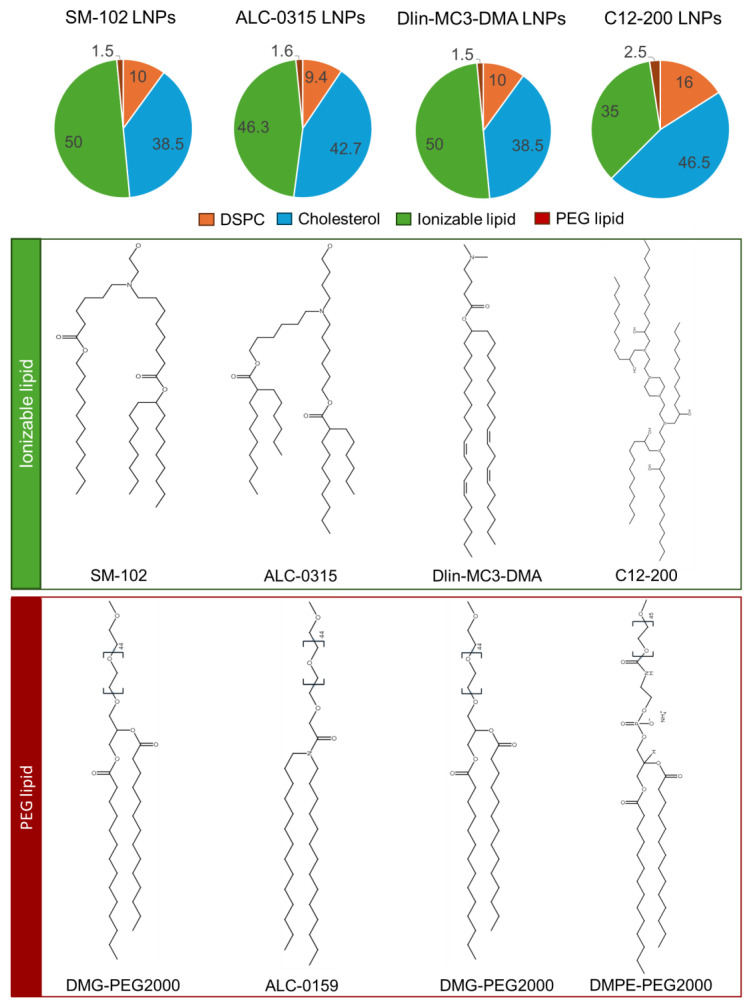
Formulation and Composition of Lipid Nanoparticles (LNPs) Investigated in this Study. The pie charts represent the lipid composition (percentage by molar ratio) of the LNP formulations, including the ionizable lipid, DSPC (phospholipid), cholesterol, and PEG-lipid. Four LNP formulations are shown, each differing in the type of ionizable lipid and/or PEG-lipid used. The ionizable lipids included were SM-102, ALC-0315, MC3, or C12-200, while the PEG-lipids vary in alkyl chain length and structure (DMG-PEG, ALC-0159, or DMPE-PEG). The molecular structures of each ionizable lipid and PEG-lipid are illustrated below the pie charts.

Therefore, within this paper, we expand on these previous studies, including the work carried out by Escalona-Ryao et al., and further explore in vitro–in vivo correlations. To achieve this, we systematically evaluate the physicochemical properties of 4 different LNP formulations using the ionisable lipids, SM-102, ALC-0315, DLin-MC3-DMA (MC3), and C12-200 (Figure 1). We assess the in vitro performance using three commonly used cell lines (HEK293, HeLa and THP-1) and evaluate in vivo outcomes in commonly adopted protein and vaccine expression mouse studies to understand further the challenges in bridging the in vitro-in vivo gap.

## 2. Materials and Methods

### 2.1. Materials

The ionisable lipids Heptadecan-9-yl 8-((2-hydroxyethyl)[6-oxo-6-(undecyloxy)hexyl]amino)octanoate (SM-102), 4-hydroxybutyl)azanediyl]di(hexane-6,1-diyl) bis(2-hexyldecanoate (ALC-0315), (6Z,9Z,28Z,31Z)-Heptatriaconta-6,9,28,31-tetraen-19-yl 4-(dimethylamino)butanoate (D-Lin-MC3-DMA), and 1,1′-[[2-[4-[2-[2-[bis(2-hydroxydodecyl)amino]ethylamino]ethyl]-1-piperazinyl]ethyl]imino]bis-2-dodecanol (C12-200) were purchased from Broadpharm (San Diego, CA, USA). The polyethylene glycol (PEG) lipids Methoxypolyethyleneglycoloxy(2000)-N,N-ditetradecylacetamide (ALC-0159), 1,2-dimyristoyl-sn-glycero-3-phosphoethanolamine-N-[methoxy(polyethylene glycol)-2000] (DMPE-PEG2000), and 1,2-dimyristoylrac-glycero-3-methoxypolyethylene glycol-2000 (DMG-PEG2000) were obtained from Avanti Polar Lipids (Alabaster, AL, USA). The phospholipid 1,2-distearoyl-sn-glycero-3-phosphocholine (DSPC) was sourced from Lipoid (Ludwigshafen, Germany). Cholesterol (Chol) and polyadenylic acid (PolyA) were acquired from Merck Life Science (Hertfordshire, UK). Phosphate-buffered saline (PBS, pH 7.4) tablets were obtained from Oxoid Ltd. (Basingstoke, UK). Other reagents, including Tris Hydrochloride (pH 7.4), Sodium Citrate buffer (pH 4), Amicon Spin Column^®^ 100K, ethanol (EtOH), sodium acetate, and sodium chloride, were obtained from Fisher Scientific (Loughborough, UK). The One-Glo Luciferase Assay System and D-Luciferin+K (VivoGlo Luciferin) were purchased from Promega Ltd. (Chilworth, UK). Messenger RNA (EZ Cap Firefly Luciferase mRNA, R1018-APE) and EZ Cap OVA mRNA (R1028-APE) were sourced from Stratech Scientific (St Thomas’ Place, Cambridgeshire, UK), while CPI provided Green Lantern mRNA. Dulbecco’s Modified Eagle’s Medium (DMEM), Minimal Essential Medium (MEM), fetal bovine serum (FBS), sodium pyruvate, and penicillin/streptomycin were acquired from Gibco Technologies. RPMI 1640 Medium was obtained from Corning (Flintshire, UK).

All solvents and reagents were of analytical grade, and Milli-Q water was produced using an in-house purification system.

### 2.2. Formulation of Lipid Nanoparticles (LNPs)

Four LNP formulations (Figure 1) were prepared using the NanoAssemblr Ignite from Precision NanoSystem Inc (Vancouver, BC, Canada). Lipid stocks were prepared individually in ethanol. All formulations were prepared at a final mRNA concentration of 70 µg/mL. Citrate buffer was used as the aqueous phase to prepare LNPs containing SM-102, ALC-0315 or MC3, whilst sodium acetate pH 4 50 mM and sodium chloride pH 4, 100 mM was used to prepare C12-200 LNPs and an N/P ratio of 8 was used for all formulations. The flow rate ratio (aqueous to solvent) of 3:1 and a total flow rate of 15 mL/min was used. DiLC was added to the lipid phase for cell uptake studies at a concentration of 1% of the total molarity, and PolyA was used as a surrogate for the mRNA. For biodistribution studies, 1% of the total molarity of DiD was added to the LNPs.

### 2.3. Removal of Ethanol Content and Buffer Exchange

LNPs were purified using 100 kDa centrifugal spin columns and were centrifuged at 2000× *g* at 20 °C until the desired volume of the LNPs was recovered. Before purification, all formulations were diluted 1 in 40 in 10 mM Tris/300 mM Sucrose.

### 2.4. LNP Characterisation: Particle Size, Polydispersity and Zeta Potential

Particle size (Z-average diameter), polydispersity index (PDI), and zeta potential (ZP) were measured using dynamic light scattering (DLS) with a Zetasizer Ultra (Malvern Panalytical Ltd., Worcestershire, UK) equipped with a 633 nm laser and a detection angle of 173°. For particle sizing and polydispersity measurements, LNPs were diluted in filtered (0.22 µm) 10 mM Tris buffer to a final concentration of 0.1 mg/mL. Zeta potential measurements were conducted with samples diluted to a final Tris concentration of 0.5 mM. The refractive index (RI) and viscosity of the dispersant (Tris) were set at 1.34 and 1.0037 cP, respectively, while the material absorbance and RI were 0.001 and 1.45, respectively. Data acquisition was performed using Zetasizer Software v.7.11 (Malvern Panalytical Ltd., Worcestershire, UK).

### 2.5. Quantification of mRNA Encapsulation Efficiency

The RiboGreen Assay was performed to determine the encapsulation efficiency of mRNA following the manufacturer’s instructions. Briefly, 50 µL of each sample was added to a 96-well black plate, with separate wells containing either 0.1% *w*/*v* TE-Triton X-100 (+Triton) or no Triton (−Triton) to assess total and unencapsulated mRNA, respectively. The plate was incubated at 37 °C for 15 min, after which the RiboGreen fluorescent dye was added to both the standards and LNP samples. The dye was added at a 1:200 dilution for +Triton wells and a 1:500 dilution for −Triton wells. Fluorescence intensity was measured using a GloMax^®^ Discover Microplate Reader at an excitation wavelength of 480 nm and an emission wavelength of 520 nm. Encapsulation efficiency (%) was calculated using the standard curve generated from the +Triton and −Triton conditions according to Equation (1).

(1) $$\mathrm{Encapsulation}\;\mathrm{efficiency}\;\left(\mathrm{EE}\%\right)=\frac{\mathrm{Total}\;\mathrm{mRNA}-\mathrm{Unencapsulated}\;\mathrm{mRNA}\;}{\mathrm{Total}\;\mathrm{mRNA}}\times 100\%$$

### 2.6. Cell Culture and In Vitro Assays

HEK293 cells were maintained in MEM, HeLa cells in DMEM, and THP-1 cells in RPMI 1649, with all media supplemented with 10% fetal bovine serum (FBS), 1% L-glutamine, 1% penicillin/streptomycin, 1% sodium pyruvate, and 2.5 µg/mL amphotericin B. The cells were incubated at 37 °C in a 5% CO_2_ atmosphere. Cytotoxicity and uptake assays for PolyA-loaded LNPs containing 1% mole DilC18 were performed using HEK293, HeLa, and THP-1 cell lines. Briefly, 100 µL of cells at 80% confluence were seeded into 96-well plates at densities of 1.5 × 10^4^ cells/well for HEK293, 0.8 × 10^4^ cells/well for HeLa, and 3 × 10^4^ cells/well for THP-1, followed by incubation at 37 °C, 5% CO_2_ for 48 h. Before seeding, THP-1 cells were differentiated with 0.2 µM phorbol 12-myristate 13-acetate (PMA). The cells were then treated with 100 µL of LNPs at concentrations ranging from 0.25 to 2 µg/mL for either 24 or 48 h. For viability assessment, the cells were incubated with 1X AlamarBlue reagent (AlamarBlue™ Cell Viability Reagent, Thermo Fisher, Paisley, UK) or treated with 10% Triton X-100 in PBS for 4 h. Fluorescence was measured at 570 nm and 595 nm to determine cell viability. LNP uptake was quantified by measuring fluorescence intensity using a GloMax^®^ Discover Microplate Reader, with a linear calibration curve generated up to 500 ng/mL (R^2^ ≥ 0.998). To assess FLuc mRNA expression, the cells were treated with LNPs at concentrations of 0.25 to 2 µg/mL for 24 or 48 h, followed by the addition of 100 µL of the One-Glo™ Luciferase Assay System (Promega, Southampton, UK). Bioluminescence was quantified by measuring total luminescence. For GFP mRNA, the cells were imaged and analysed for green fluorescent protein expression.

### 2.7. In Vivo Biodistribution and Bioluminescence Imaging of Mice Receiving mRNA-Loaded LNPs

Groups of three female BALB/c mice (6–9 weeks old) were housed under standard conditions (22 °C, 55% humidity, 12 h light/dark cycle) and provided with a standard diet ad libitum. Each mouse received a 50 µL intramuscular injection (100 µg/mL mRNA) into both quadricep muscles. Bioluminescence imaging was performed using an IVIS Spectrum (REVVITY (Wales, UK)), and data capture and analysis were conducted via Living Image software 4.8.2. mRNA expression was detected based on the bioluminescence emission of firefly luciferase (560 nm). Imaging parameters included medium binning, an f/stop of 2, and an acquisition time determined using auto-exposure settings. The mice were anaesthetised with 3% Isoflurane for induction, with anaesthesia maintained at 1% Isoflurane during imaging. The images were captured at 6, 24, and 48 h post-injection. Bioluminescence intensity was quantified by calculating the total flux (photons/second) at the injection site (region of interest). The average total flux was determined and expressed as mean ± SEM.

### 2.8. Immunisation and ELISA Analysis of Serum-Specific IgG Isotypes

Groups of three female BALB/c mice (6–9 weeks old) were immunised intramuscularly in the right thigh on days 0 and 28 with 5 μg of mRNA encoding ovalbumin (OVA) formulated in one of four LNP formulations, delivered in a 50 μL volume. Serum samples were collected from individual mice one day before immunisation, on day 27, and two weeks after the booster (day 42) to evaluate the efficacy of the formulations in eliciting serum-specific IgG isotypes (Total IgG, IgG1, and IgG2a). For antibody quantification, 96-well microtitre plates (Greiner Bio-One GmbH, Frickenhausen, Germany) were coated overnight at 4 °C with 100 μL of albumin from chicken egg white (OVA) (Merck Life Science, Hertfordshire, UK) at a concentration of 1 μg/mL in PBS (pH 9.0). The plates were washed three times with wash buffer (PBS pH 7.4 with 0.05% *v*/*v* Tween-20) and blocked with 150 μL of 4% *w*/*v* Marvel^®^ solution in PBS (pH 7.4) for 1 h at 37 °C. After three additional washes, 100 μL of serially diluted serum samples (1:800 or higher, depending on the experiment) were added to the appropriate wells and incubated for 1 h at 37 °C. Following washing, 100 μL of horseradish peroxidase (HRP)-conjugated goat anti-mouse Total IgG, IgG1, or IgG2a antibodies were added at dilutions of 1:2500 (IgG) or 1:5000 (IgG1 and IgG2a), and the plates were incubated for 1 h at 37 °C. After three washes, 100 μL of TMB substrate (Fisher Scientific, Loughborough, UK) was added to each well, and the reaction was stopped after 20 min with 50 μL of 10% aqueous sulphuric acid. Absorbance was measured at 450 nm using a Microplate Manager^®^ Device (Bio-Rad Laboratories, Inc., Hercules, CA, USA), and the mean endpoint titre ± standard deviation (SD) was calculated for each group.

### 2.9. Ethics Statement

All animal experiments and procedures were conducted in compliance with UK Home Office regulations and approved by the University of Strathclyde Animal Welfare and Ethical Review Board under project licence number PPL PP1650440. BALB/c mice were bred and housed in the Biological Procedures Unit at the University of Strathclyde, Glasgow. The experimental design and reporting followed the ARRIVE guidelines.

### 2.10. Statistical Analysis

The results are represented as the mean ± SD of at least *n* = 3 independent batches for physicochemical analysis and mean ± SEM of at least *n* = 2 independent batches for biological analysis in vitro. For mRNA expression and vaccine studies, 3 mice per group were used, and studies were undertaken in duplicate, giving a total of 6 mice per formulation. ANOVA tests were used to assess statistical significance, with Tukey’s post-ad hoc test to compare the four LNP formulations (*p*-value of less than 0.05). The Kruskal–Wallis test was carried out to compare immune responses.

## 3. Results

### 3.1. Physicochemical Attributes of mRNA-LNPs

The CQAs of LNPs across four formulations (SM102, ALC-0135, MC3, and C12-200) with three different mRNA encapsulated are shown in Table 1. The results show that the LNPs have similar properties, with minor variations highlighting their consistent performance. Particle sizes were within the expected range for LNPs, averaging around 85–100 nm, with slightly smaller (70–85 nm) sizes observed for C12-200. Polydispersity Index (PDI) values indicated overall uniformity, with low variability across formulations (PDI < 0.2). Zeta potentials were generally near neutral. Encapsulation efficiency (%EE) was high across all formulations, typically exceeding 90%, demonstrating the ability of these LNPs to encapsulate mRNA effectively, as would be expected.

### 3.2. Establishing Standard In Vitro Protocols for Testing LNPs

**Figure 2:**
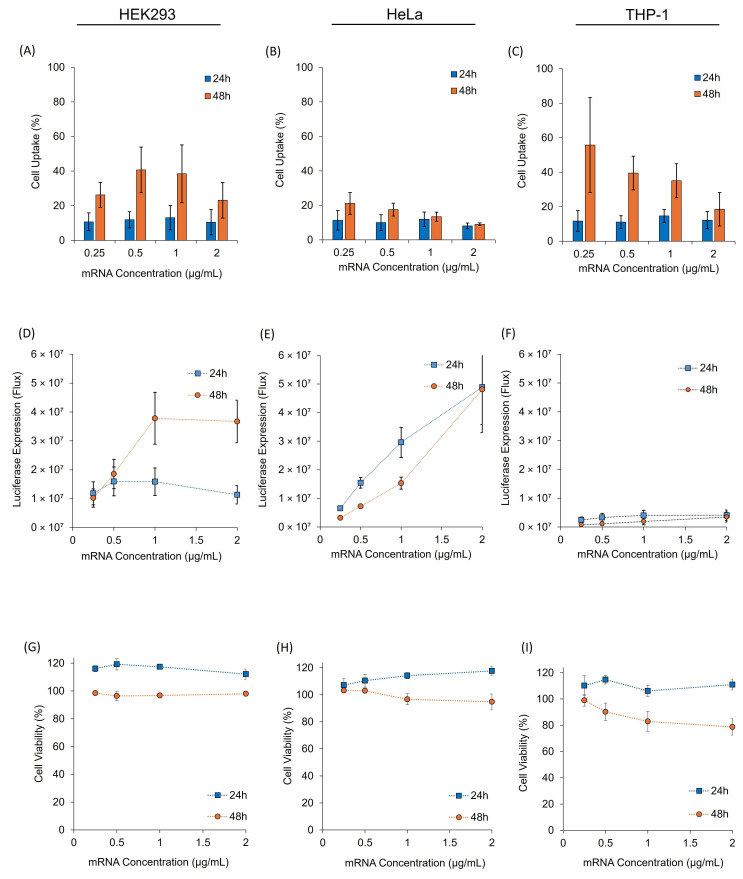
In Vitro Performance of SM102 LNPs Across Three Different Cell Lines at 24 and 48 h. SM-102 LNP cell uptake in (**A**) HEK293, (**B**) HeLa and (**C**) THP-1 cell lines. mRNA expression (luciferase) in (**D**) HEK293, (**E**) HeLa and (**F**) THP-1. Cell viability after treatment with mRNA-LNPs in (**G**) HEK293, (**H**) HeLa and (**I**) THP-1. In all experiments, the cells were incubated with 0.25, 0.5, 1 and 2 µg/mL mRNA. Results represent mean ± SEM, n = 3 independent batches.

Given that all LNP formulations exhibited similar physicochemical properties, the next step was to evaluate their functional attributes in vitro and assess their cell uptake, transfection efficiency, and cell viability. Initially, SM102 LNPs were investigated in three cell lines: HEK293, HeLa and THP-1 cells (differentiated into macrophages before testing). These cell lines are commonly used as they are readily available and can be easily expanded [7]. The cells were treated with 0.25 to 2 µg/mL of mRNA-LNPs and incubated for 24 and 48 h to determine the optimal incubation time for cell uptake, LNP expression and cell viability (Figure 2). SM-102 was selected for the initial in vitro optimisation due to its widespread use and well-established performance in both in vitro and in vivo models. Cell uptake studies were performed using LNPs tagged with a fluorescent dye, DiIC. The cells were lysed with 10% TritonX-100 at each time point, and the fluorescence was measured.

All three cell lines showed modest increases in cell uptake at 48 h compared with 24 h, with HEK293 and THP-1 cells showing higher cell uptake than HeLa cells (Figure 2A–C). When considering mRNA expression, our studies showed that both the HEK293 (Figure 2D) and HeLa (Figure 2E) cells had higher protein (FLuc) expression than the THP-1 cells (Figure 2F). HEK293 cells showed significantly higher levels of expression after a 48 h compared to a 24 h incubation period (*p* < 0.05), with a dose-dependent increase in expression observed when the mRNA concentration increased from 0.25 to 1 µg/mL mRNA (Figure 2D). HeLa cells tended to show higher expression levels at 24 h compared to 48 h, and a dose-dependent increase in expression was observed as the mRNA concentration increased from 0.25 to 2 μg/mL mRNA (Figure 2E). However, with THP-1 cells, there was no significant difference (*p* > 0.05) in the expression across the time points or concentrations, and overall expression was low (Figure 2F). When considering cell uptake and expression, we saw little correlation. However, it is important to note that our approach was based on a high-throughput 96 well-plate format to assess LNP uptake. It does not differentiate between internalisation and surface adsorption, which can impact data interpretation. A more detailed approach to investigate cell uptake could be undertaken using confocal or flow cytometry studies. However, given the generally low uptake noted in Figure 2, this was not investigated further. Cell viability studies show no notable difference in cell viability across all mRNA concentrations tested in all three cell lines, with a slight decrease in viability when the cells were incubated for 48 h compared to 24 h (Figure 2G–I). Based on these results, we selected 48 h incubation for the HEK293 cells and 24 h incubation for both HeLa and THP-1 cell lines for further experiments.

### 3.3. Comparative In Vitro Evaluation of Four LNP Formulations Across Multiple Cell Lines

After optimising in vitro parameters, four LNP formulations (Table 1) were tested using the refined protocol. The cells were treated with LNPs at mRNA concentrations ranging from 0.25 to 2 μg/mL and incubated for the duration established by SM-102 optimisation (Figure 2).

**Figure 3:**
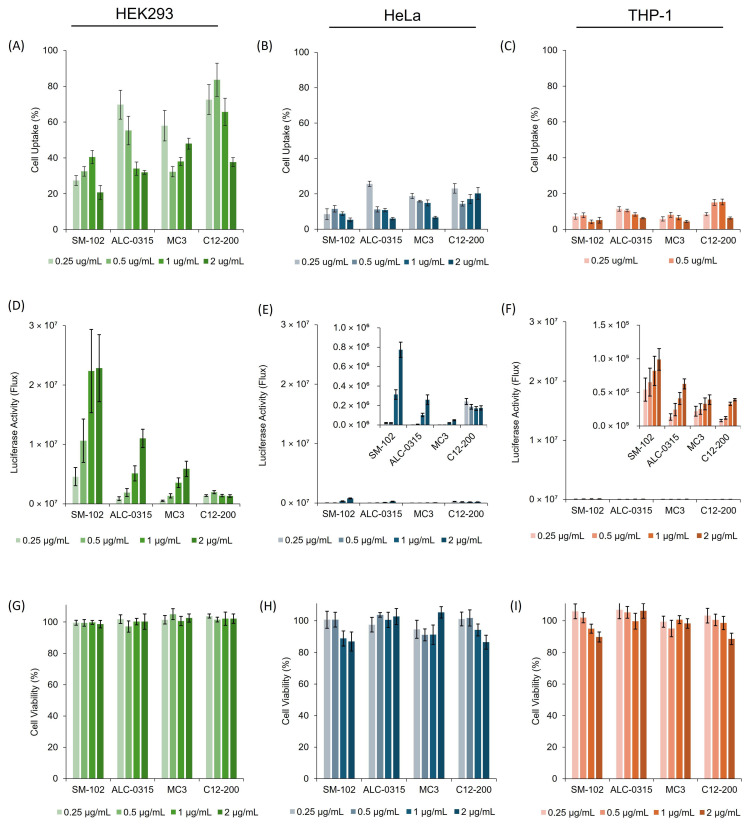
In vitro comparison of four different LNP formulations (Figure 1) containing the different ionisable lipids, SM-102, ALC-0315, MC3 and C12-200, across three different cell lines (HEK293, HeLa and THP-1). Cell uptake of four different LNP formulations was measured using (**A**) HEK293, (**B**) HeLa, and (**C**) THP-1 cell lines. mRNA expression (luciferase) for the four LNPs in (**D**) HEK293, (**E**) HeLa, and (**F**) THP-1 cells. Cell viability after treatment with mRNA-LNPs in (**G**) HEK293, (**H**) HeLa, and (**I**) THP-1. In all experiments, the cells were incubated with 0.25, 0.5, 1, and 2 µg/mL mRNA. The results represent mean ± SEM, n = 3 independent batches.

Figure 3 shows that HEK293 cells had an overall higher uptake for all formulations than HeLa and THP-1 cells (Figure 3A–C) and higher protein expression levels (Figure 3D–F). Cell viability remained high across all concentrations, formulations, and cell lines (>90%; Figure 3G–I). When comparing the four formulations in HEK293 cells at the highest mRNA concentration (2 µg/mL), MC3 showed significantly higher (*p* < 0.05) uptake than ALC-0315 and SM-102, with SM-102 having significantly lower uptake than the three other formulations. However, when measuring the cell expression, SM-102 has significantly higher expression (*p* < 0.05) than ALC-0315, MC3 and C12-200 (which had no significant difference between them) (Figure 3D–F). Indeed, SM-102 LNPs promoted significantly higher (*p* < 0.05) expression across all cell lines (Figure 3D–F). Dose–response relationships were observed for SM-102, ALC-0315, and MC3 LNPs in all cell lines; however, C12-200 LNPs did not show a dose–response in HEK293 or HeLa cells (Figure 3D–F).

**Figure 4:**
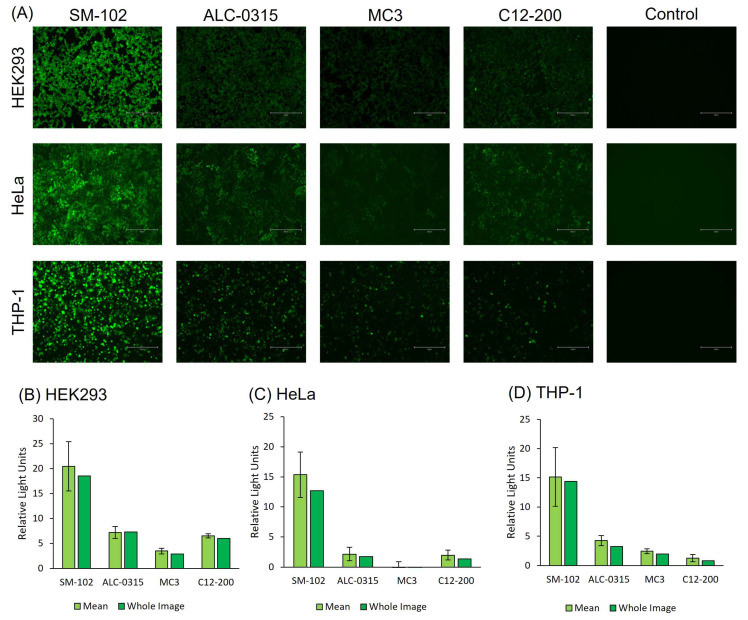
(**A**) Cell images of HEK293, HeLa and THP-1 cells treated with 2 µg/mL GFP-mRNA. GFP expression was quantified using ImageJ for (**B**) HEK293, (**C**) HeLa and (**D**) THP-1 cells. These results are representative of three biological replicates from 1 LNP batch.

The four LNP formulations were also tested for expression using GFP-mRNA (Figure 4). The GFP-mRNA formulations were incubated in all three cell lines for either 24 h (HeLa and THP-1 cell lines) or 48 h (HEK293 cells), and images were taken for each formulation at the same concentration and using the same light settings on the microscope. The results in Figure 4 align with the luciferase cell expression profile in Figure 3; SM102 LNPs consistently have the highest cell expression levels across all three cell lines, followed by ALC, MC3 and C12-200 LNPs.

### 3.4. Comparative In Vivo Expression Studies in a Murine Model

**Figure 5:**
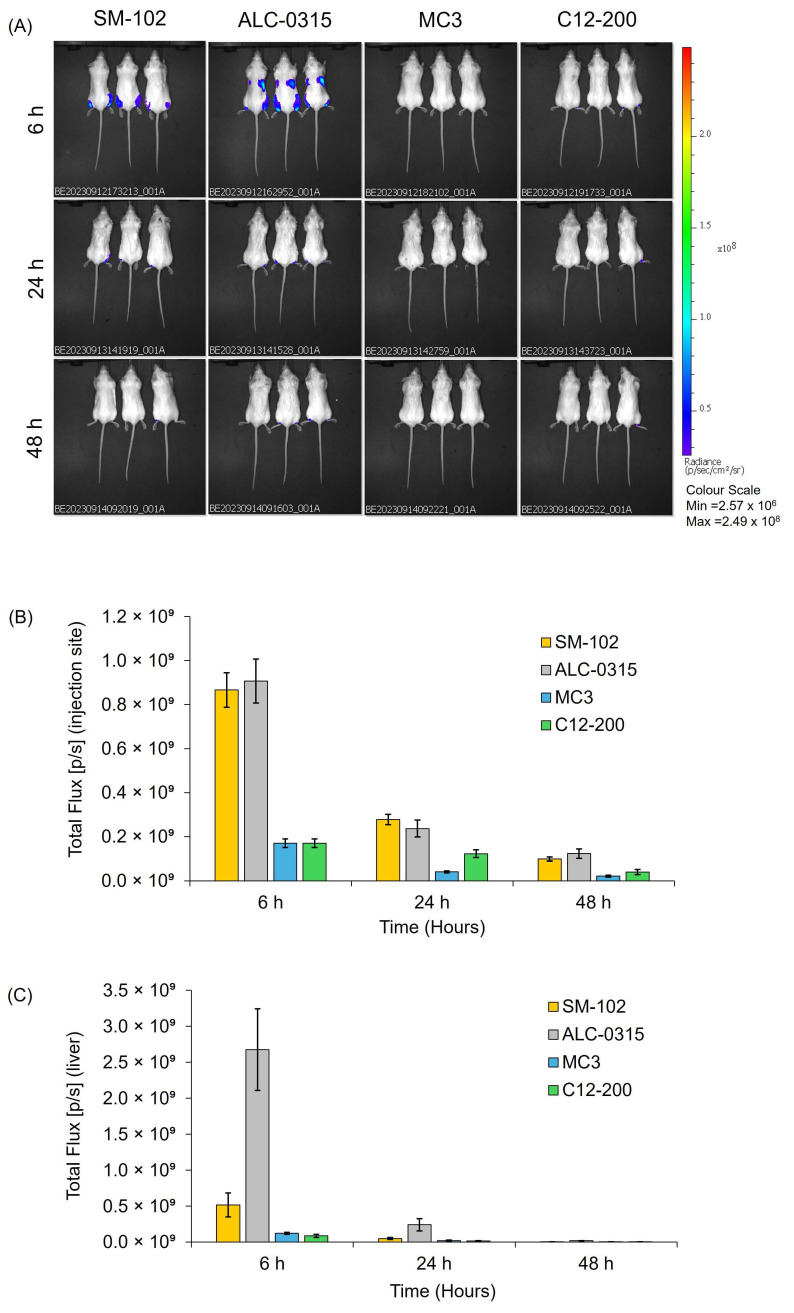
In Vivo luciferase expression after Fluc-mRNA LNP intramuscular injection in female BALB/c mice. The mice were administered 5 μg of mRNA encapsulated in LNPs per injection. (**A**) Representative IVIS bioluminescence images were captured at selected time points following mRNA-LNP injection. (**B**) Bioluminescence intensity at the injection site was measured at 6, 24, and 48 h. (**C**) Bioluminescence signal in the liver was also assessed at these time points. The data are expressed by the mean ± SEM and represent n = 2 independent batches of each formulation of LNPs (six mice per group split over two independent studies).

The in vivo expression of mRNA-LNPs was evaluated using BALB/c mice, which received intramuscular injections of 5 µg per dose in both hind legs. The four LNP formulations (Table 1) were compared for their expression profiles (Figure 5) and biodistribution (Supplementary Figure S1).

The in vivo mRNA expression images (Figure 5A) show that SM-102 and ALC-0315 formulations promoted the highest overall luminescent signal in mice at the injection site and liver, with the strongest expression seen at early time points (6 h) and a gradual reduction over subsequent imaging sessions. Meanwhile, MC3 and C12-200 LNPs promoted modest expression that reduced over time (Figure 5A). Comparing the luciferase expression promoted by the four different LNPs at the injection site (Figure 5B) demonstrates high levels of expression for both SM-102 and ALC-0135 LNP formulations (with no significant difference between them, *p* > 0.05). In contrast, significantly (*p* < 0.05) lower expression was promoted by both MC3 and C12-200 LNPs compared to both SM-102 and ALC-0315 LNPs. A similar trend in expression profile at the injection site is shown at 24 and 48 h, but at lower levels (Figure 5B). However, ALC-0315 LNPs had significantly higher liver expression than the other four LNPs (*p* < 0.05), followed by SM-102 LNPs, which had a 5-fold lower expression at 6 h (Figure 5C). After this first time point, the expression levels were not significantly different between the four formulations (*p* > 0.05). When looking at the biodistribution (Supplementary Figure S1), there is no significant difference between the biodistribution of the 4 LNPs at 6, 24 and 48 h.

### 3.5. Immunisation Study

**Figure 6:**
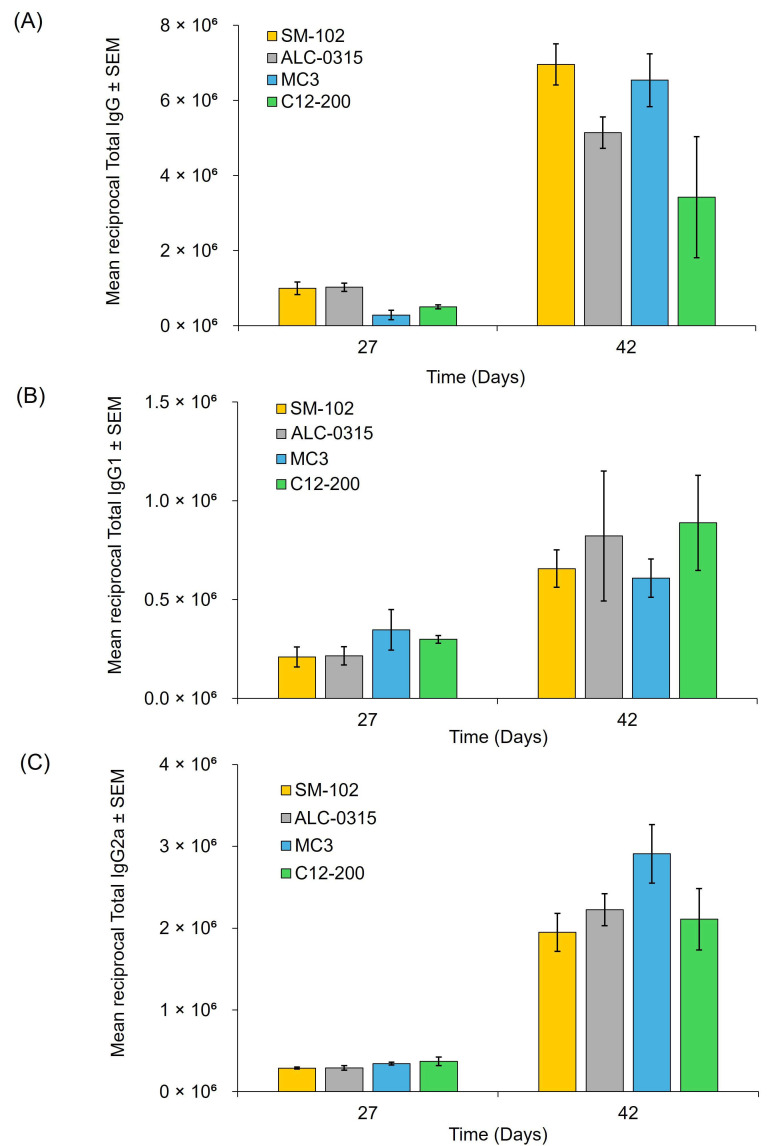
Antibody Responses in BALB/c Mice Following Immunisation with OVA mRNA-LNPs. Antibody responses were assessed in female BALB/c mice immunised with 5 μg of OVA mRNA encapsulated in one of four LNP formulations on days 0 and 28. Serum samples were collected via tail bleeding before the first dose and after both the first and second doses. (**A**) Total anti-OVA IgG endpoint titres induced by OVA mRNA LNPs. (**B**) IgG1 levels targeting OVA protein. (**C**) Anti-OVA IgG2a endpoint titres generated by OVA mRNA LNPs. The data are expressed by the mean ± SEM and represent n = 2 independent batches of each formulation of LNPs (six mice per group split over two independent studies).

To study the vaccine efficacy of the mRNA-LNPs, BALB/c mice were immunised with 5 µg of OVA mRNA encapsulated in the four different LNPs. Mice received intramuscular injections following a prime-boost regimen, with the initial dose administered on day 0 and a booster on day 28. Serum samples were collected one day prior to each injection and again two weeks after the booster (day 42). Antibody endpoint titres were measured using ELISA (Figure 6).

The results demonstrate that the highest responses are seen at day 42, as expected, and antibody subtype responses are in the order IgG > IgG2a > IgG1 (Figure 6). Across the three antibody subtypes (IgG, IgG1 and IgG2a), there are no significant differences (*p* > 0.05) in antibody responses to the encoded antigen between the groups that received the four different LNP formulations (Figure 6), suggesting that all LNPs have similar potency in terms of stimulating an immune response.

## 4. Discussion

The performance of mRNA-LNPs is governed by their capacity to encapsulate, protect, and deliver mRNA to target cells. Once in vivo, additional factors such as interactions with the biological environment, biodistribution, immune activation, and inherent adjuvanticity must be considered [2]. Successful mRNA-LNP delivery is a multi-step process involving cell surface attachment, internalisation, and endosomal escape to enable ribosomal protein expression [3] and at each stage, a portion of the mRNA payload can be lost [5]. As a result, in vitro models, often combined with physicochemical screening, are commonly used for early assessment of LNP efficacy and help guide down-selection toward preclinical evaluations. However, numerous studies report little or modest correlations between in vitro and in vivo protein expression [5,6].

To further investigate how these discrepancies might impact formulation selection for in vivo testing, four commonly used LNP formulations based on SM-102, ALC-0315, MC3, and C12-200 were chosen to study in vitro and in vivo correlations both in terms of protein expression and vaccine performance in mouse models. All formulations were produced by microfluidics, resulting in similar attributes (particle size < 100 nm, PDI < 0.1, and encapsulation efficiency > 80%) (Table 1). It is well-recognised that the prominent factor that drives LNP characteristics is the manufacturing process, and manipulation of manufacturing parameters, including flow rate ratio and total flow rate, can be used to control the physicochemical attributes of LNPs [8,9,10,11]. Therefore, despite the different formulations, all LNPs were similar in the physicochemical attributes measured.

Following their characterisation, the next step in the development of LNPs is to test their efficacy, and cell culture models represent an important pre-clinical assessment for LNP delivery and efficacy in vitro [12]. In our investigations, in vitro studies were carried out in immortalised (HEK293 and HeLa) and immune cells (THP-1) to allow for initial assessment of mRNA-LNP cell viability and transfection potency. These cell lines were selected as they are commonly used for mRNA-LNP development [13,14,15]. Protocols were first optimised with SM-102 LNPs (Figure 2) and then applied to compare all four formulations (Figure 3 and Figure 4). The SM-102 LNP formulation was selected for the optimisation of the in vitro protocols due to its widespread use. However, it is also important to acknowledge that the best protocol for SM-102 may not be the best for other ionisable LNP formulations. Across both Fluc and GFP mRNA payloads, SM-102 LNPs consistently yielded the highest expression, followed by ALC-0315, MC3, and C12-200 LNPs in all three cell lines (Figure 3 and Figure 4). HEK293 displayed the highest expression among the cell lines, followed by HeLa and THP-1 (Figure 2, Figure 3 and Figure 4). Generally, in all cell lines tested, cell uptake did not correlate with expression (Figure 3).

The major mechanism for the internalisation of LNPs is mediated through a clathrin-mediated pathway due to the structural similarities between LNPs and chylomicrons (lipoprotein particles composed of phospholipids, triglycerides, cholesterol and protein). This pathway has been demonstrated to depend on the LNP CQAs, including the charge on the LNP surfaces [16]. Neutral LNPs have shown an increased preference for cellular uptake compared with highly positive or negatively charged LNPs due to electrostatic repulsion in the presence of phospholipids and proteoglycans in the cell membrane [17,18]. Cell uptake of LNPs is also influenced by LNP interactions with endogenous proteins such as apolipoprotein E (ApoE), which can direct LNPs to hepatocytes via receptor-mediated endocytosis by low-density lipoprotein receptors (LDL-R) [2,3]. This may explain why HEK293 cells (known to produce ApoE and well-known for their high transfection efficiency) display high expression in our studies, whereas HeLa cells, which do not produce ApoE (though they express ApoE receptors [19]), showed comparatively lower expression. Differentiated THP-1 cells can also produce ApoE. However, the expression pattern is heterogeneous, with approximately 5% of the cells overexpressing ApoE, which subsequently results in apoptosis of the cells [20]. However, C12-200 has been reported to undergo cell uptake via an ApoE-independent, micropinocytosis pathway [21]. Love et al. also investigated the mechanism by which C12-200 LNPs are internalised, specifically using HeLa cells [22]. In their study, C12-200 particles encapsulating siRNA were incubated in the presence of labelled cargoes known to enter cells by different endocytic pathways. Their results suggested that C12-200 was internalised via a macropinocytosis mechanism due to membrane ruffling, and actin rearrangement was observed in the cells.

Following cellular uptake, efficient protein expression depends on successful endosomal escape, which is influenced by the pKa and chemical structure of ionizable lipids [23]. Cone-shaped ionisable lipids, characterised by a small head group and broader tail, disrupt the organisation of cylindrical lipids in the endosomal membrane. This incompatibility leads to membrane destabilisation, facilitating mRNA release into the cytosol for subsequent translation. However, SM-102, ALC-0315, and MC3 are designed to become protonated at roughly the same acidic endosomal pH. This similarity in pKa should facilitate equivalent levels of endosomal membrane disruption and mRNA release into the cytoplasm. Equally, SM-102 and ALC-0315 share core design principles optimised for efficient intracellular delivery.

The in vivo performance of the four LNP formulations was evaluated using two mRNA payloads: FLuc (for protein expression) and OVA (for antibody responses). The biodistribution of the labelled LNPs with DiR was measured as a fluorescence signal intensity (Supplementary Figure S1) using the IVIS spectrum, and the results showed that the signal remained relatively constant across the 48 h study with no signal detected in the liver. This may be attributed to a combination of factors including tissue optical properties and dye aggregation induced quenching [16,24]. We observed that in vitro protein expression did not correlate with in vivo protein expression (both SM102 and ALC-315 LNPs performed well; Figure 5) or with immune responses (all LNP formulations promoted similar antibody responses; Figure 6). While most studies on LNP development focus on their physicochemical characteristics (e.g., [25,26]) and many have examined IVIVC in terms of protein expression (e.g., [27,28]), here we have systematically tracked the four commonly used LNP formulations through standard characterisation, in vitro and in vivo expression, and in vivo immune responses. We demonstrated that in vitro studies do not predict in vivo protein expression, and in vivo protein expression does not correlate with vaccine efficacy.

In general, developing predictive IVIVC is crucial for biological research; however, while tissue culture aims to mimic in vivo conditions, the lack of tissue architecture and heterogeneous cell populations often fail to replicate innate tissue functions [29]. As a result, they differ from their in vivo counterparts because of foreign serum exposure and static flow [5]. Using primary cells can help overcome these limitations. For instance, Whitehead et al. demonstrated improved IVIVC using primary hepatocytes instead of immortalised cells for siRNA lipidoid silencing [30]. The disadvantage of using primary cell lines is that the inherent expression profile only lasts 1–2 days post-isolation, and culturing these cells is technical, time-consuming and expensive and thus does not lend itself to high-throughput screening [30]. Consequently, immortalised cell lines remain more practical. In developing an IVIVC assay, there is a balance between providing optimal comparison conditions and tailoring the protocol for each specific LNP, which would undermine the goal of developing a universal assay framework. However, further optimisation of these in vitro protocols (potentially back-correlating with in vivo data) is an important consideration when addressing IVIVC.

Both our results and other studies [31] show that in vitro and in vivo protein expression does not always correlate with vaccine efficacy. The similar antibody responses (IgG, IgG1, IgG2a) induced by the four different LNP formulations could be resulting from the LNPs offering an adjuvant effect potentially in combination with a threshold effect: once the antigen expression reaches a minimum level necessary to stimulate an immune response, further increases in transfection efficiency may not translate into higher antibody titers. Given their immune-stimulatory properties, LNPs should be considered more than mere excipients. The ionisable lipid in mRNA-LNP vaccines can induce IL-6 production, eliciting potent helper T cell and B cell responses that were independent of the RNA-sensing pathways [32]. Because the mRNA is non-immunogenic, the LNP itself provides the adjuvant activity [33]. A recent study by Chaudhary et al. [34] showed that amine headgroups in ionizable lipids drive immune responses by binding TLR4 and CD1d receptors, thereby promoting a type-1 T-helper-cell-biassed response and increasing specific immunoglobulins and pro-inflammatory cytokines. These signals can also inhibit anti-poly(ethylene glycol) IgM production, preserving LNP delivery efficacy. In our vaccine study (Figure 6), different ionisable lipids produced similar antibody responses in a standard BALB/c model, suggesting that either the protocol does not discriminate effectively between formulations at the doses we tested or the TLR4/CD1d responses do not impact antibody outcomes.

## 5. Conclusions

Developing predictive IVIVC for mRNA-LNPs remains challenging due to the complexity of transfection mechanisms, endosomal escape, and immune interactions. In vitro assays (commonly performed in immortalised cell lines) offer rapid, cost-effective insights but do not always predict in vivo performance reliably. Our results demonstrate that bridging the gap between carefully controlled in vitro conditions and complex in vivo biology remains challenging. The oversimplification of in vitro models fails to fully replicate the in vivo environment, ultimately leading to discrepancies when transitioning between animal models and human applications. Furthermore, different ionizable lipids may behave differently depending on the optimisation of the in vitro protocol. Additionally, there is a major gap when investigating immune system interactions. While LNPs are designed to deliver mRNA effectively, their interaction with the immune system can vary significantly between in vitro and in vivo settings. These findings confirm that current commonly used in vitro models cannot be relied upon for LNP formulation testing and down-selection, and more accurate protocols (which could consider back-correlation of in vivo data) are needed to support reliable pre-clinical development of LNPs.

## Figures and Tables

**Table 1:** LNP physicochemical characteristics of LNPs encapsulating either Fluc, GFP or OVA mRNA. LNPs were produced at a FRR 3:1 and TFR 15 mL/min using microfluidics. Results represent n = 4 independent batches for Fluc mRNA and n = 3 for GFP and OVA mRNA formulations.

	Size (nm)			PDI			Zeta (mV)			%EE		
mRNA	Fluc	GFP	OVA	Fluc	GFP	OVA	Fluc	GFP	OVA	Fluc	GFP	OVA
SM102	97 ± 7	98 ± 5	99 ± 1	0.07 ± 0.02	0.07 ± 0.02	0.07 ± 0.02	9 ± 4	8 ± 2	8 ± 5	94 ± 2	98 ± 0	95 ± 1
ALC-0135	85 ± 6	90 ± 11	90 ± 2	0.09 ± 0.02	0.11 ± 0.03	0.11 ± 0.02	−2 ± 3	2 ± 3	−2 ± 5	85 ± 5	95 ± 2	90 ± 2
MC3	93 ± 3	101 ± 6	99 ± 2	0.05 ± 0.02	0.06 ± 0.02	0.11 ± 0.02	7 ± 2	7 ± 1	0 ± 2	92 ± 1	98 ± 1	93 ± 3
C12-200	73 ± 2	73 ± 10	85 ± 9	0.08 ± 0.03	0.13 ± 0.03	0.14 ± 0.02	−4 ± 4	−1 ± 1	−7 ± 3	98 ± 2	97 ± 2	97 ± 3

## Data Availability

https://doi.org/10.15129/b286909d-5b90-4efc-81e4-982ae3684919, accessed on 10 March 2025.

## Supplementary Materials

The following supporting information can be downloaded at: https://www.mdpi.com/article/10.3390/vaccines13040339/s1.
