# Supplementary material for: Exploring the Challenges of Lipid Nanoparticle Development: The In Vitro–In Vivo Correlation Gap
Source: Vaccines (Basel). 2025 Mar 21;13(4):339. doi: 10.3390/vaccines13040339 (PMC12031360; doi:10.3390/vaccines13040339)
Supplement: Supplementary file 1 [file vaccines-13-00339-s001.zip › vaccines-3479500-supplementary.pdf]

Figure S1.

Exploring the Challenges of Lipid Nanoparticle Development: The In Vitro–In Vivo Correlation Gap

Sarah Lindsay[1], Muattaz Hussain[1], Burcu Binici[1] and Yvonne Perrie[1,*]

[1]Strathclyde Institute of Pharmacy and Biomedical Sciences, University of Strathclyde, 161 Cathedral Street, G4 0RE, Glasgow, UK
*Correspondence: Yvonne.perrie@strath.ac.uk

a)

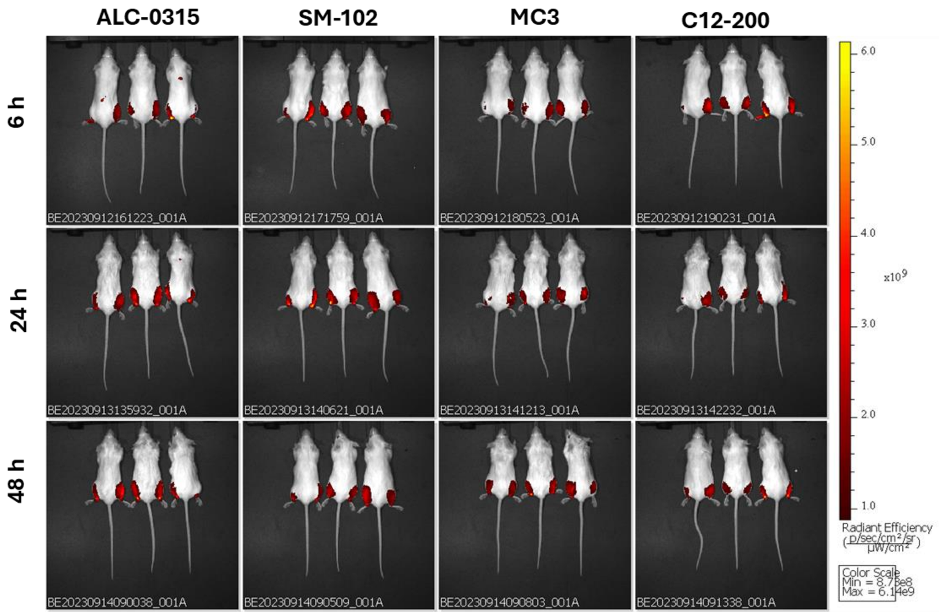

b)

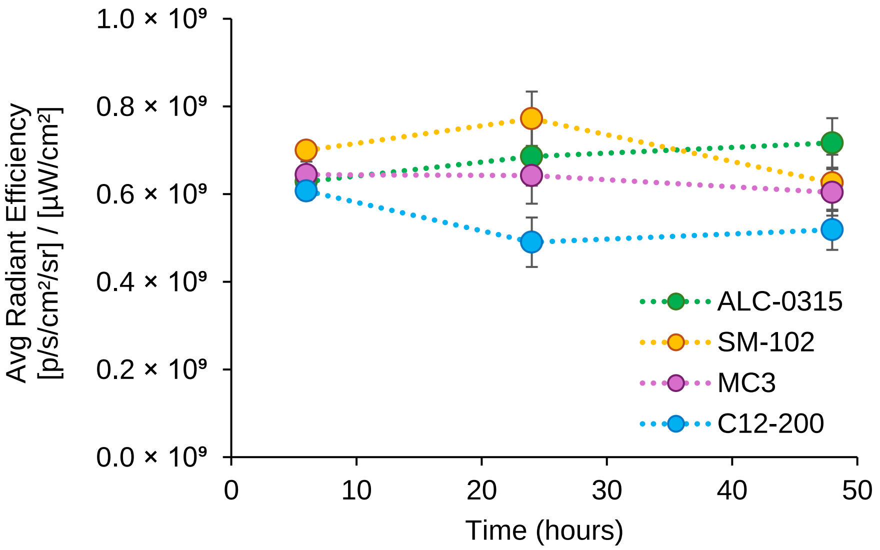

Figure S1. The biodistribution profile of DiR labelled Fluc-mRNA LNPs at 6, 24 and 48 h post IM injection. a) The quantification of DiR intensity in the injection site over the time and b) Representative IVIS image. LNPs were labelled with 5 µg of DiR labelled Fluc mRNA-LNPs and imaged under DiR filter on IVIS across the time. Result represent mean ± SEM (a total of 6 mice split over 2 independent studies). The representative image is from one of the replicates.
